# Oxide of Zinc and Eugenol as a Covering to Pulps

**Published:** 1900-10

**Authors:** S. B. Luckie

**Affiliations:** Chester, Pa.


### OXIDE OF ZINC AND EUGENOL AS A COVERING TO PULPS.[1]

BY S. B. LUCKIE, D.D.S., CHESTER, PA.

That which makes the mixture of oxide of zinc and eugenol of value is that it possesses the properties of a good protector to the pulp, a therapeutic agent and a filling-material combined, and can be used as either one or all, if desirable, in the same case. It makes a good covering for arsenous acid, as it can from the peripheries of the cavity be domed over the application, thus avoiding pressure, and, being a soothing obtundent, adds to the possibility of lessening or preventing pain, so likely to follow arsenical dressings.

The evidence of its having therapeutic properties comes from clinical observation alone. Teeth that have ached from pulpitis,

---

[1] Read before The New York Institute of Stomatology, May 1, 1900.

after having the pain relieved, if filled with it, will often remain comfortable for months, extending into years in some cases.

Eugenol, as given by Merck, is antiseptic, anti-tubercular, and a local anæsthetic. Oxide of zinc is a tonic, antispasmodic, and astringent; in general medicine it is used as an exsiccant on excoriated surfaces, either by sprinkling or in the form of an ointment.

What therapeutic advantage, other than to make a mechanical agent to protect from external irritation, is gained by mixing the two drugs I have not been able to find out. If, however, the properties of each be retained after the mixture is made, it is hard to conceive of more desirable properties for a lining or as an intermediary in deep cavities.

While its virtues are many, I do not wish it understood that they are extolled to the same degree as is done for proprietary cements for pulp covering and dressing; it will not in all cases maintain comfort in all the stages of pulpitis, nor does it do away with all pulp devitalization and extirpation, but in the hands of the careful and conscientious practitioner it will aid in giving comfort after filling in those cases of sensitive dentine and of deep-seated caries, and very greatly reduce the number of cases of pulpitis and pericementitis that occur in filled teeth. It can be used to fill deciduous teeth where it would not be wise to prepare the cavity for a metal filling, and is often preferable to gutta-percha, as its use is more possible to maintain comfort and prevent pulp and peridental complications. It makes a good trial filling where caries have approached very near the pulp, and if no complications follow, when worn away it can be covered with amalgam or gold.

In those teeth where the pulps had died under a filling of oxide of zinc and eugenol, the first case is to be presented with the pulp putrescent and foul smelling, the contents of the chamber and canals always being dry, and there being no exhibition of peridental irritation. Of course, it is to be understood these were cases seen before the filling had wasted sufficiently to allow the fluids of the mouth to enter the pulp-chamber.

As an intermediary under zinc phosphate fillings, it will prevent the phosphoric acid from irritating the pulp. In all cavities of much depth on the occlusal surfaces of bicuspids and molars it should precede the filling, and in approximal cavities a pad of it should be placed on the region overlying the pulp, the mix being made quite thick to hasten the setting, and the cavity walls be covered with a quick-drying lining and filled with whatever material is designated.

To enumerate, the cement made from oxide of zinc and eugenol is useful to fill cavities with exquisitively sensitive dentine for a period of time elapsing from a few weeks to months, as a trial filling, as an intermediary in deep cavities, as a covering for pulp- and canal-dressings, and as a non-removable canal-filling. As a canal-filling it can be used with advantage on account of its slow setting, allowing more than ample time for its introduction. The method adopted for canal-filling is to pump it in with Swiss broaches until all possible parts of the canal are apparently reached by it, when a cone of temporary stopping is placed in the canal and pressure made towards the apex.

With an experience of about six years, it has been a valuable aid in the treatment of teeth and in preventing the discomfort from thermal changes that so often follow their filling. I am not unconscious, however, that it might be improved by the addition of other antiseptics; indeed, it has often occurred that a series of bacteriological experiments might be conducted with advantage to prove the value of different intermediary used between filling and dentine, or as pulp-caps.

Aristol or hydronaphthol can be mixed with oxide of zinc, in about equal quantity, and, if mixed with eugenol, a cement with about the same degree of hardness is obtained, though clinically I have obtained no advantage from the addition.

